# 4-Methyl-5-phenyl-1*H*-pyrazol-3-ol

**DOI:** 10.1107/S1600536810022828

**Published:** 2010-06-18

**Authors:** Tara Shahani, Hoong-Kun Fun, R. Venkat Ragavan, V. Vijayakumar, S. Sarveswari

**Affiliations:** a X-ray Crystallography Unit, School of Physics, Universiti Sains Malaysia, 11800 USM, Penang, Malaysia; b Organic Chemistry Division, School of Advanced Sciences, VIT University, Vellore 632 014, India

## Abstract

The title compound, C_10_H_10_N_2_O, crystallizes with two independent mol­ecules in the asymmetric unit, having closely comparable geometries. The dihedral angles between the 1*H*-pyrazole and benzene rings in the two mol­ecules are 39.57 (14) and 41.95 (13)°. The two mol­ecules are each connected to neighbouring mol­ecules by pairs of inter­molecular O—H⋯N hydrogen bonds, forming dimers with *R*_2_^2^(8) ring motifs. These dimers are further linked into *R*_4_^4^(10) ring motifs by inter­molecular N—H⋯O hydrogen bonds, forming chains along [101]. The crystal structure is further stabilized by a C—H⋯π inter­action.

## Related literature

For the biological activity of 4-methyl-3-phenyl-1*H*-pyrazol-5-ol, see: Brogden (1986 ▶); Gursoy *et al.* (2000 ▶); Ragavan *et al.* (2009 ▶, 2010 ▶); Watanabe *et al.* (1984 ▶); Kawai *et al.* (1997 ▶); Wu *et al.* (2002 ▶). For related structures, see: Shahani *et al.* (2009 ▶, 2010*a* ▶,*b* ▶,*c* ▶). For hydrogen-bond motifs, see: Bernstein *et al.* (1995 ▶). For bond-length data, see: Allen *et al.* (1987 ▶). For the stability of the temperature controller used for the data collection, see: Cosier & Glazer (1986 ▶).
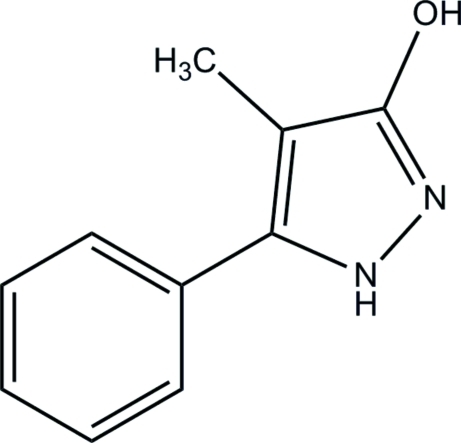


## Experimental

### 

#### Crystal data

C_10_H_10_N_2_O
*M* *_r_* = 174.20
Monoclinic, 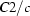

*a* = 26.4082 (19) Å
*b* = 11.0972 (8) Å
*c* = 14.1245 (10) Å
β = 118.996 (1)°
*V* = 3620.4 (4) Å^3^
*Z* = 16
Mo *K*α radiation
μ = 0.09 mm^−1^
*T* = 100 K
0.35 × 0.14 × 0.08 mm

#### Data collection

Bruker APEXII DUO CCD area-detector diffractometer
Absorption correction: multi-scan (*SADABS*; Bruker, 2009 ▶) *T*_min_ = 0.970, *T*_max_ = 0.993
19166 measured reflections
5255 independent reflections
2907 reflections with *I* > 2σ(*I*)
*R*_int_ = 0.049

#### Refinement

*R*[*F* ^2^ > 2σ(*F* ^2^)] = 0.059
*wR*(*F* ^2^) = 0.204
*S* = 1.13
5255 reflections
243 parameters
H atoms treated by a mixture of independent and constrained refinement
Δρ_max_ = 0.33 e Å^−3^
Δρ_min_ = −0.26 e Å^−3^

### 

Data collection: *APEX2* (Bruker, 2009 ▶); cell refinement: *SAINT* (Bruker, 2009 ▶); data reduction: *SAINT*; program(s) used to solve structure: *SHELXTL* (Sheldrick, 2008 ▶); program(s) used to refine structure: *SHELXTL*; molecular graphics: *SHELXTL*; software used to prepare material for publication: *SHELXTL* and *PLATON* (Spek, 2009 ▶).

## Supplementary Material

Crystal structure: contains datablocks global, I. DOI: 10.1107/S1600536810022828/is2561sup1.cif

Structure factors: contains datablocks I. DOI: 10.1107/S1600536810022828/is2561Isup2.hkl

Additional supplementary materials:  crystallographic information; 3D view; checkCIF report

## Figures and Tables

**Table 1:** Hydrogen-bond geometry (Å, °) *Cg*1 is the centroid of the C1*B*–C6*B* benzene ring.

*D*—H⋯*A*	*D*—H	H⋯*A*	*D*⋯*A*	*D*—H⋯*A*
O1*A*—H1*OA*⋯N2*A*^i^	0.83	1.85	2.673 (2)	171
O1*B*—H1*OB*⋯N2*B*^ii^	0.83	1.84	2.670 (2)	177
N1*B*—H1*NB*⋯O1*A*^iii^	1.00 (3)	1.85 (3)	2.836 (3)	171 (3)
N1*A*—H1*NA*⋯O1*B*^iv^	0.97 (3)	1.88 (3)	2.844 (2)	173 (2)
C10*A*—H10*C*⋯*Cg*1^v^	0.96	2.77	3.575 (3)	142

Symmetry codes: (i) 
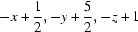
; (ii) 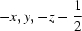; (iii) 
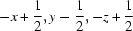
; (iv) 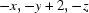; (v) 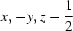.

**Fig. 1.**
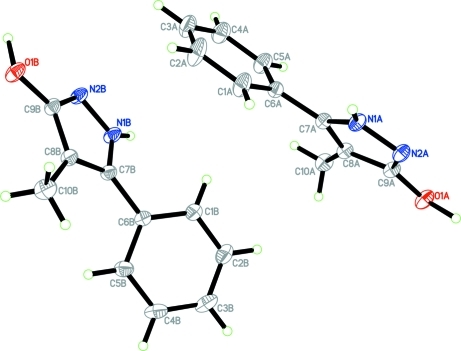
The molecular structure of the title compound, showing 20% probability displacement ellipsoids and the atom numbering scheme.

**Fig. 2.**
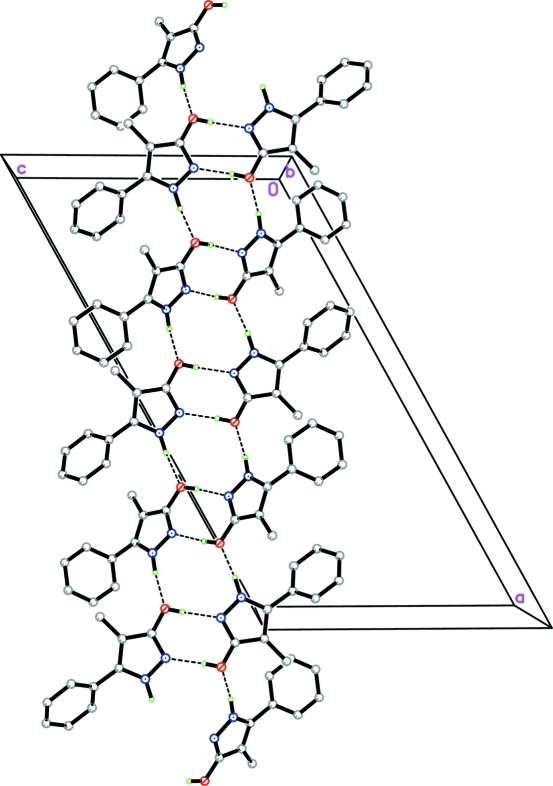
The crystal packing of the title compound, viewed approximately along the b axis, showing a one-dimensional chain.

## supplementary crystallographic information

### Comment

Pyrazolone derivatives have a broad spectrum of biological activities being used as analgesic, antipyretic and anti-inflammatory therapeutical drugs (Brogden, 1986; Gursoy *et al.*, 2000). A class of new compounds with the pyrazolone moiety was synthesized and reported for their antibacterial and antifungal activities by Ragavan *et al.* (2009, 2010). A new pyrazolone derivative, edaravone (3-methyl-1-phenyl-2-pyrazoline-5-one), is being used as a drug in clinical practice for brain ischemia (Watanabe *et al.*, 1984; Kawai *et al.*, 1997) and the same has been found to be effective against myocardial ischemia (Wu *et al.*, 2002).

There are two independent molecules (A and B) in the asymmetric unit (Fig. 1). The maximum deviations in 1*H*-pyrazole ring (N1/N2/C7–C9) for molecules A and B are 0.006 (2) and 0.011 (2) Å, respectively, at atoms C6A and C6B. The dihedral angles formed between the 1*H*-pyrazole ring and benzene ring in molecules A and B are 39.57 (14) and 41.95 (13)°, respectively. The bond lengths (Allen *et al.*, 1987) and angles are within normal ranges and comparable to those closely related structures (Shahani *et al.*, 2009, 2010*a*–*c*).

In the crystal packing (Fig. 2), pairs of intermolecular O1A—H1OA···N2A and O1B—H1OB···N2B hydrogen bonds (Table 1) form dimers with neighbouring molecules, generating *R*_2_^2^(8) ring motifs (Bernstein *et al.*, 1995). These dimers are further linked into *R*_4_^4^(10) ring motifs by additional intermolecular N1A—H1NA···O1B and N1B—H1NB···O1A hydrogen bonds (Table 1), forming one dimensional chains along the [101] direction. The crystal structure is further stabilized by C—H···π interaction (Table 1), involving the C1B—C6B benzene ring (centroid *Cg*1).

### Experimental

The compound 4-methyl-5-phenyl-1-*H*-pyrazol-3-ol has been synthesized using the method available in the literature (Ragavan *et al.*, 2009, 2010) and recrystallized using the ethanol (white solid). *m.p.* 278.5–493 K.

### Refinement

The H atoms bound to O atoms were located in a difference map and constrained to ride with their parent atoms, with *U*_iso_(H) = 1.5*U*_eq_(O) (O—H = 0.83 Å). The H atoms bound to N atoms were located in a difference map and were refined freely [refined N—H lengths, 1.00 (3) and 0.97 (2) Å]. All other H atoms were positioned geometrically (C—H = 0.93–0.96 Å], with *U*_iso_(H) = 1.2 or 1.5*U*_eq_(C).

### Crystal data

**Table:**

C_10_H_10_N_2_O	*F*(000) = 1472
*M**_r_* = 174.20	*D*_x_ = 1.278 Mg m^−^^3^
Monoclinic, *C*2/*c*	Mo *K*α radiation, λ = 0.71073 Å
Hall symbol: -C 2yc	Cell parameters from 3052 reflections
*a* = 26.4082 (19) Å	θ = 3.3–27.2°
*b* = 11.0972 (8) Å	µ = 0.09 mm^−^^1^
*c* = 14.1245 (10) Å	*T* = 100 K
β = 118.996 (1)°	Block, colourless
*V* = 3620.4 (4) Å^3^	0.35 × 0.14 × 0.08 mm
*Z* = 16	

### Data collection

**Table:**

Bruker APEXII DUO CCD area-detector diffractometer	5255 independent reflections
Radiation source: fine-focus sealed tube	2907 reflections with *I* > 2σ(*I*)
graphite	*R*_int_ = 0.049
φ and ω scans	θ_max_ = 30.0°, θ_min_ = 2.0°
Absorption correction: multi-scan (*SADABS*; Bruker, 2009)	*h* = −37→28
*T*_min_ = 0.970, *T*_max_ = 0.993	*k* = −15→13
19166 measured reflections	*l* = −19→19

### Refinement

**Table:**

Refinement on *F*^2^	Primary atom site location: structure-invariant direct methods
Least-squares matrix: full	Secondary atom site location: difference Fourier map
*R*[*F*^2^ > 2σ(*F*^2^)] = 0.059	Hydrogen site location: inferred from neighbouring sites
*wR*(*F*^2^) = 0.204	H atoms treated by a mixture of independent and constrained refinement
*S* = 1.13	*w* = 1/[σ^2^(*F*_o_^2^) + (0.0985*P*)^2^] where *P* = (*F*_o_^2^ + 2*F*_c_^2^)/3
5255 reflections	(Δ/σ)_max_ = 0.001
243 parameters	Δρ_max_ = 0.33 e Å^−^^3^
0 restraints	Δρ_min_ = −0.26 e Å^−^^3^

### Special details

**Table:**

Experimental. The crystal was placed in the cold stream of an Oxford Cyrosystems Cobra open-flow nitrogen cryostat (Cosier & Glazer, 1986) operating at 100.0 (1) K.
Geometry. All e.s.d.'s (except the e.s.d. in the dihedral angle between two l.s. planes) are estimated using the full covariance matrix. The cell e.s.d.'s are taken into account individually in the estimation of e.s.d.'s in distances, angles and torsion angles; correlations between e.s.d.'s in cell parameters are only used when they are defined by crystal symmetry. An approximate (isotropic) treatment of cell e.s.d.'s is used for estimating e.s.d.'s involving l.s. planes.
Refinement. Refinement of *F*^2^ against ALL reflections. The weighted *R*-factor *wR* and goodness of fit *S* are based on *F*^2^, conventional *R*-factors *R* are based on *F*, with *F* set to zero for negative *F*^2^. The threshold expression of *F*^2^ > σ(*F*^2^) is used only for calculating *R*-factors(gt) *etc*. and is not relevant to the choice of reflections for refinement. *R*-factors based on *F*^2^ are statistically about twice as large as those based on *F*, and *R*- factors based on ALL data will be even larger.

### Fractional atomic coordinates and isotropic or equivalent isotropic displacement parameters (Å^2^)

**Table:**

	*x*	*y*	*z*	*U*_iso_*/*U*_eq_	
O1A	0.30484 (5)	1.15234 (15)	0.48624 (10)	0.0513 (4)	
H1OA	0.3021	1.1865	0.5356	0.077*	
N1A	0.17011 (7)	1.19165 (16)	0.26752 (12)	0.0419 (4)	
N2A	0.21137 (6)	1.22060 (17)	0.36985 (11)	0.0416 (4)	
C1A	0.09436 (9)	1.0463 (3)	0.07114 (18)	0.0643 (7)	
H1AA	0.0772	1.0599	0.1142	0.077*	
C2A	0.06077 (11)	1.0113 (3)	−0.0353 (2)	0.0835 (10)	
H2AA	0.0211	1.0019	−0.0634	0.100*	
C3A	0.08503 (12)	0.9902 (3)	−0.09985 (19)	0.0704 (8)	
H3AA	0.0621	0.9665	−0.1715	0.084*	
C4A	0.14342 (12)	1.0042 (3)	−0.05821 (19)	0.0676 (7)	
H4AA	0.1603	0.9906	−0.1017	0.081*	
C5A	0.17726 (10)	1.0384 (3)	0.04793 (17)	0.0605 (6)	
H5AA	0.2170	1.0462	0.0758	0.073*	
C6A	0.15335 (8)	1.06126 (19)	0.11381 (14)	0.0412 (4)	
C7A	0.18926 (8)	1.10453 (19)	0.22525 (14)	0.0386 (4)	
C8A	0.24504 (8)	1.07398 (19)	0.30334 (14)	0.0406 (4)	
C9A	0.25656 (7)	1.1493 (2)	0.39179 (14)	0.0404 (4)	
C10A	0.28424 (9)	0.9785 (2)	0.30003 (17)	0.0526 (5)	
H10A	0.2662	0.9409	0.2300	0.079*	
H10B	0.2914	0.9190	0.3545	0.079*	
H10C	0.3202	1.0143	0.3135	0.079*	
O1B	−0.05992 (5)	0.70768 (16)	−0.21038 (10)	0.0515 (4)	
H1OB	−0.0528	0.7049	−0.2616	0.077*	
N1B	0.08261 (7)	0.69465 (18)	−0.01528 (12)	0.0468 (4)	
N2B	0.04080 (6)	0.70277 (17)	−0.12057 (12)	0.0455 (4)	
C1B	0.14424 (9)	0.7478 (2)	0.21872 (16)	0.0564 (6)	
H1BA	0.1524	0.8034	0.1787	0.068*	
C2B	0.17998 (11)	0.7390 (3)	0.32909 (18)	0.0725 (8)	
H2BA	0.2123	0.7887	0.3631	0.087*	
C3B	0.16837 (12)	0.6580 (3)	0.38904 (18)	0.0708 (8)	
H3BA	0.1925	0.6531	0.4635	0.085*	
C4B	0.12108 (10)	0.5843 (3)	0.33917 (17)	0.0630 (7)	
H4BA	0.1134	0.5285	0.3797	0.076*	
C5B	0.08471 (9)	0.5923 (2)	0.22872 (16)	0.0520 (5)	
H5BA	0.0524	0.5426	0.1954	0.062*	
C6B	0.09620 (8)	0.67397 (19)	0.16757 (14)	0.0409 (4)	
C7B	0.05896 (8)	0.68061 (19)	0.04958 (14)	0.0400 (4)	
C8B	−0.00068 (8)	0.6800 (2)	−0.01460 (14)	0.0411 (5)	
C9B	−0.00976 (8)	0.69654 (19)	−0.12047 (14)	0.0406 (4)	
C10B	−0.04620 (9)	0.6615 (3)	0.01754 (18)	0.0598 (6)	
H10D	−0.0283	0.6528	0.0947	0.090*	
H10E	−0.0679	0.5901	−0.0167	0.090*	
H10F	−0.0717	0.7298	−0.0047	0.090*	
H1NB	0.1235 (11)	0.687 (3)	0.0001 (19)	0.073 (8)*	
H1NA	0.1316 (11)	1.224 (2)	0.2420 (18)	0.064 (7)*	

### Atomic displacement parameters (Å^2^)

**Table:**

	*U*^11^	*U*^22^	*U*^33^	*U*^12^	*U*^13^	*U*^23^
O1A	0.0298 (6)	0.0806 (12)	0.0370 (7)	0.0088 (6)	0.0112 (5)	−0.0066 (7)
N1A	0.0321 (7)	0.0554 (11)	0.0348 (7)	0.0049 (7)	0.0134 (6)	−0.0018 (7)
N2A	0.0299 (7)	0.0569 (11)	0.0336 (7)	0.0034 (7)	0.0120 (6)	−0.0038 (7)
C1A	0.0429 (11)	0.093 (2)	0.0548 (12)	−0.0142 (12)	0.0221 (10)	−0.0223 (12)
C2A	0.0463 (13)	0.126 (3)	0.0661 (15)	−0.0210 (15)	0.0174 (12)	−0.0364 (17)
C3A	0.0739 (17)	0.079 (2)	0.0488 (12)	−0.0139 (14)	0.0223 (12)	−0.0220 (12)
C4A	0.0755 (16)	0.082 (2)	0.0516 (12)	−0.0043 (14)	0.0357 (12)	−0.0183 (12)
C5A	0.0478 (11)	0.0854 (19)	0.0496 (11)	−0.0037 (12)	0.0245 (10)	−0.0106 (12)
C6A	0.0393 (9)	0.0436 (12)	0.0394 (9)	−0.0020 (8)	0.0180 (8)	−0.0021 (8)
C7A	0.0342 (8)	0.0461 (12)	0.0385 (9)	−0.0015 (8)	0.0199 (7)	−0.0007 (8)
C8A	0.0330 (8)	0.0515 (13)	0.0383 (9)	0.0012 (8)	0.0181 (7)	−0.0002 (8)
C9A	0.0295 (8)	0.0549 (13)	0.0363 (9)	0.0031 (8)	0.0157 (7)	0.0020 (8)
C10A	0.0426 (10)	0.0601 (15)	0.0530 (11)	0.0109 (10)	0.0214 (9)	−0.0005 (10)
O1B	0.0298 (6)	0.0862 (12)	0.0357 (7)	0.0068 (7)	0.0135 (6)	−0.0007 (6)
N1B	0.0305 (8)	0.0740 (14)	0.0338 (7)	−0.0012 (8)	0.0139 (6)	0.0038 (7)
N2B	0.0291 (7)	0.0713 (13)	0.0316 (7)	0.0007 (7)	0.0113 (6)	0.0029 (7)
C1B	0.0556 (12)	0.0620 (16)	0.0418 (10)	−0.0101 (11)	0.0160 (9)	0.0013 (10)
C2B	0.0650 (15)	0.088 (2)	0.0436 (12)	−0.0171 (14)	0.0097 (11)	−0.0075 (12)
C3B	0.0685 (16)	0.102 (2)	0.0352 (10)	0.0073 (15)	0.0196 (11)	0.0036 (12)
C4B	0.0648 (14)	0.0853 (19)	0.0455 (11)	0.0113 (13)	0.0320 (11)	0.0194 (11)
C5B	0.0494 (11)	0.0626 (15)	0.0466 (10)	0.0015 (10)	0.0253 (9)	0.0098 (10)
C6B	0.0398 (9)	0.0474 (12)	0.0349 (8)	0.0043 (8)	0.0176 (8)	0.0034 (8)
C7B	0.0369 (9)	0.0481 (12)	0.0351 (9)	−0.0006 (8)	0.0174 (8)	0.0021 (8)
C8B	0.0338 (9)	0.0519 (13)	0.0384 (9)	−0.0004 (8)	0.0180 (8)	0.0009 (8)
C9B	0.0308 (8)	0.0524 (13)	0.0363 (9)	0.0002 (8)	0.0145 (7)	−0.0011 (8)
C10B	0.0405 (11)	0.0886 (19)	0.0561 (12)	0.0052 (11)	0.0280 (10)	0.0070 (12)

### Geometric parameters (Å, °)

**Table:**

O1A—C9A	1.326 (2)	O1B—C9B	1.323 (2)
O1A—H1OA	0.8273	O1B—H1OB	0.8317
N1A—C7A	1.356 (3)	N1B—C7B	1.345 (2)
N1A—N2A	1.3612 (19)	N1B—N2B	1.359 (2)
N1A—H1NA	0.97 (2)	N1B—H1NB	1.00 (3)
N2A—C9A	1.337 (2)	N2B—C9B	1.338 (2)
C1A—C2A	1.380 (3)	C1B—C2B	1.380 (3)
C1A—C6A	1.381 (3)	C1B—C6B	1.384 (3)
C1A—H1AA	0.9300	C1B—H1BA	0.9300
C2A—C3A	1.365 (4)	C2B—C3B	1.368 (4)
C2A—H2AA	0.9300	C2B—H2BA	0.9300
C3A—C4A	1.367 (4)	C3B—C4B	1.368 (4)
C3A—H3AA	0.9300	C3B—H3BA	0.9300
C4A—C5A	1.375 (3)	C4B—C5B	1.383 (3)
C4A—H4AA	0.9300	C4B—H4BA	0.9300
C5A—C6A	1.378 (3)	C5B—C6B	1.383 (3)
C5A—H5AA	0.9300	C5B—H5BA	0.9300
C6A—C7A	1.470 (2)	C6B—C7B	1.470 (2)
C7A—C8A	1.388 (2)	C7B—C8B	1.385 (2)
C8A—C9A	1.409 (3)	C8B—C9B	1.407 (3)
C8A—C10A	1.498 (3)	C8B—C10B	1.491 (3)
C10A—H10A	0.9600	C10B—H10D	0.9600
C10A—H10B	0.9600	C10B—H10E	0.9600
C10A—H10C	0.9600	C10B—H10F	0.9600
			
C9A—O1A—H1OA	115.1	C9B—O1B—H1OB	106.7
C7A—N1A—N2A	111.00 (15)	C7B—N1B—N2B	110.74 (15)
C7A—N1A—H1NA	130.4 (15)	C7B—N1B—H1NB	130.8 (14)
N2A—N1A—H1NA	117.7 (14)	N2B—N1B—H1NB	117.7 (14)
C9A—N2A—N1A	105.76 (15)	C9B—N2B—N1B	106.09 (15)
C2A—C1A—C6A	120.2 (2)	C2B—C1B—C6B	120.0 (2)
C2A—C1A—H1AA	119.9	C2B—C1B—H1BA	120.0
C6A—C1A—H1AA	119.9	C6B—C1B—H1BA	120.0
C3A—C2A—C1A	120.9 (2)	C3B—C2B—C1B	120.7 (2)
C3A—C2A—H2AA	119.5	C3B—C2B—H2BA	119.7
C1A—C2A—H2AA	119.5	C1B—C2B—H2BA	119.7
C2A—C3A—C4A	119.3 (2)	C4B—C3B—C2B	119.8 (2)
C2A—C3A—H3AA	120.3	C4B—C3B—H3BA	120.1
C4A—C3A—H3AA	120.3	C2B—C3B—H3BA	120.1
C3A—C4A—C5A	120.1 (2)	C3B—C4B—C5B	120.2 (2)
C3A—C4A—H4AA	119.9	C3B—C4B—H4BA	119.9
C5A—C4A—H4AA	119.9	C5B—C4B—H4BA	119.9
C4A—C5A—C6A	121.3 (2)	C4B—C5B—C6B	120.3 (2)
C4A—C5A—H5AA	119.4	C4B—C5B—H5BA	119.9
C6A—C5A—H5AA	119.4	C6B—C5B—H5BA	119.9
C5A—C6A—C1A	118.19 (18)	C1B—C6B—C5B	119.03 (18)
C5A—C6A—C7A	120.97 (18)	C1B—C6B—C7B	120.16 (18)
C1A—C6A—C7A	120.81 (18)	C5B—C6B—C7B	120.79 (18)
N1A—C7A—C8A	107.63 (16)	N1B—C7B—C8B	108.12 (15)
N1A—C7A—C6A	121.31 (16)	N1B—C7B—C6B	120.11 (16)
C8A—C7A—C6A	131.04 (18)	C8B—C7B—C6B	131.71 (17)
C7A—C8A—C9A	104.45 (17)	C7B—C8B—C9B	104.41 (16)
C7A—C8A—C10A	129.14 (17)	C7B—C8B—C10B	129.08 (17)
C9A—C8A—C10A	126.32 (17)	C9B—C8B—C10B	126.47 (17)
O1A—C9A—N2A	122.24 (17)	O1B—C9B—N2B	121.95 (16)
O1A—C9A—C8A	126.60 (17)	O1B—C9B—C8B	127.43 (17)
N2A—C9A—C8A	111.16 (15)	N2B—C9B—C8B	110.60 (15)
C8A—C10A—H10A	109.5	C8B—C10B—H10D	109.5
C8A—C10A—H10B	109.5	C8B—C10B—H10E	109.5
H10A—C10A—H10B	109.5	H10D—C10B—H10E	109.5
C8A—C10A—H10C	109.5	C8B—C10B—H10F	109.5
H10A—C10A—H10C	109.5	H10D—C10B—H10F	109.5
H10B—C10A—H10C	109.5	H10E—C10B—H10F	109.5
			
C7A—N1A—N2A—C9A	−0.7 (2)	C7B—N1B—N2B—C9B	1.5 (2)
C6A—C1A—C2A—C3A	0.3 (5)	C6B—C1B—C2B—C3B	−0.2 (4)
C1A—C2A—C3A—C4A	0.0 (5)	C1B—C2B—C3B—C4B	0.4 (5)
C2A—C3A—C4A—C5A	0.4 (5)	C2B—C3B—C4B—C5B	−0.7 (4)
C3A—C4A—C5A—C6A	−1.2 (4)	C3B—C4B—C5B—C6B	0.8 (4)
C4A—C5A—C6A—C1A	1.4 (4)	C2B—C1B—C6B—C5B	0.2 (4)
C4A—C5A—C6A—C7A	−176.4 (2)	C2B—C1B—C6B—C7B	−178.2 (2)
C2A—C1A—C6A—C5A	−1.0 (4)	C4B—C5B—C6B—C1B	−0.5 (3)
C2A—C1A—C6A—C7A	176.8 (3)	C4B—C5B—C6B—C7B	178.0 (2)
N2A—N1A—C7A—C8A	0.7 (2)	N2B—N1B—C7B—C8B	−0.3 (2)
N2A—N1A—C7A—C6A	−177.51 (17)	N2B—N1B—C7B—C6B	−177.91 (18)
C5A—C6A—C7A—N1A	138.9 (2)	C1B—C6B—C7B—N1B	39.6 (3)
C1A—C6A—C7A—N1A	−38.9 (3)	C5B—C6B—C7B—N1B	−138.8 (2)
C5A—C6A—C7A—C8A	−38.9 (3)	C1B—C6B—C7B—C8B	−137.3 (2)
C1A—C6A—C7A—C8A	143.3 (2)	C5B—C6B—C7B—C8B	44.2 (3)
N1A—C7A—C8A—C9A	−0.5 (2)	N1B—C7B—C8B—C9B	−0.9 (2)
C6A—C7A—C8A—C9A	177.5 (2)	C6B—C7B—C8B—C9B	176.3 (2)
N1A—C7A—C8A—C10A	176.4 (2)	N1B—C7B—C8B—C10B	177.0 (2)
C6A—C7A—C8A—C10A	−5.6 (4)	C6B—C7B—C8B—C10B	−5.9 (4)
N1A—N2A—C9A—O1A	−179.23 (17)	N1B—N2B—C9B—O1B	176.63 (19)
N1A—N2A—C9A—C8A	0.4 (2)	N1B—N2B—C9B—C8B	−2.1 (2)
C7A—C8A—C9A—O1A	179.64 (19)	C7B—C8B—C9B—O1B	−176.7 (2)
C10A—C8A—C9A—O1A	2.7 (3)	C10B—C8B—C9B—O1B	5.3 (4)
C7A—C8A—C9A—N2A	0.1 (2)	C7B—C8B—C9B—N2B	1.9 (2)
C10A—C8A—C9A—N2A	−176.89 (19)	C10B—C8B—C9B—N2B	−176.1 (2)

### Hydrogen-bond geometry (Å, °)

**Table:**

Cg1 is the centroid of the C1B–C6B benzene ring.

**Table:**

*D*—H···*A*	*D*—H	H···*A*	*D*···*A*	*D*—H···*A*
O1A—H1OA···N2A^i^	0.83	1.85	2.673 (2)	171
O1B—H1OB···N2B^ii^	0.83	1.84	2.670 (2)	177
N1B—H1NB···O1A^iii^	1.00 (3)	1.85 (3)	2.836 (3)	171 (3)
N1A—H1NA···O1B^iv^	0.97 (3)	1.88 (3)	2.844 (2)	173 (2)
C10A—H10C···Cg1^v^	0.96	2.77	3.575 (3)	142

Symmetry codes: (i) −*x*+1/2, −*y*+5/2, −*z*+1; (ii) −*x*, *y*, −*z*−1/2; (iii) −*x*+1/2, *y*−1/2, −*z*+1/2; (iv) −*x*, −*y*+2, −*z*; (v) *x*, −*y*, *z*−1/2.
